# Sequential Decisions: A Computational Comparison of Observational and Reinforcement Accounts

**DOI:** 10.1371/journal.pone.0094308

**Published:** 2014-04-18

**Authors:** Nazanin Mohammadi Sepahvand, Elisabeth Stöttinger, James Danckert, Britt Anderson

**Affiliations:** Department of Psychology, University of Waterloo, Waterloo, Ontario, Canada; University of California, Davis, United States of America

## Abstract

Right brain damaged patients show impairments in sequential decision making tasks for which healthy people do not show any difficulty. We hypothesized that this difficulty could be due to the failure of right brain damage patients to develop well-matched models of the world. Our motivation is the idea that to navigate uncertainty, humans use models of the world to direct the decisions they make when interacting with their environment. The better the model is, the better their decisions are. To explore the model building and updating process in humans and the basis for impairment after brain injury, we used a computational model of non-stationary sequence learning. RELPH (Reinforcement and Entropy Learned Pruned Hypothesis space) was able to qualitatively and quantitatively reproduce the results of left and right brain damaged patient groups and healthy controls playing a sequential version of Rock, Paper, Scissors. Our results suggests that, in general, humans employ a sub-optimal reinforcement based learning method rather than an objectively better statistical learning approach, and that differences between right brain damaged and healthy control groups can be explained by different exploration policies, rather than qualitatively different learning mechanisms.

## Introduction

Humans are surprisingly efficient in making sequential decisions [1], [2]. This efficiency is remarkable because it occurs in the face of uncertainty (in fact, in the face of uncertainty about uncertainty; [3], [4]), relies on imperfect knowledge, must utilize ambiguous cues, and takes place in an environment of variable risk and non-deterministic outcomes [5], [6], [7], [8].

One plausible suggestion for how we cope with such uncertainty is that we are guided by mental models. Through our observations, and utilizing processes such as learning and heuristics [9], [10], [11], [12], we develop mental models of the processes generating a given sequence of events. These models encapsulate the rules that govern the environment, and form the basis for predicting what will happen next. When the environment changes, as it usually does, our models and their predictions will no longer match incoming information and thus we must update them [13], [14], [15].

Understanding model building and updating is also beneficial for brain-damage studies since these processes appear to be hemispherically lateralized and may provide a unitary account for understanding many of the non-spatial deficits that follow right brain injury [13],[16].

We are interested in understanding how humans build mental models through combining various learning processes, heuristics and prior beliefs. Patterns of impairment after brain injury may help to elucidate these connections. To investigate more about building and updating processes, we study sequential decision making tasks because of what they may reveal about these processes. In our prior studies of model updating in patients with brain damage we used the game Rock, Paper, Scissors (RPS; [13]). Participants played RPS as a sequential decision making task and participant performance could improve by learning an opponent's strategy. Since the computer opponent's strategy shifted, participant performance would improve if shifts in policies were detected and participant models updated. In this paper, we explore the nature of mental model building and updating for sequential decisions by emulating it with a computational model and replicating control and brain-damaged participant performances. By exploring which model components are necessary to reproduce participants' performances, and whether particular changes are consistent across different clinical groups, we can improve our account of the model building process.

Our plan for the paper is as follows: we first summarize the clinical data that formed the basis for our modelling. These data were previously reported and are briefly recounted here for convenience. We next develop two possible approaches that the participants might have taken to build proper mental models of the task: statistical learning and reinforcement learning approaches. First, we review the particular sequence learning model we used (Entropy Learned Pruned Hypothesis space; ELPH; [17]) and the assumptions behind it. Our expectation, based on an intuition that something like statistical learning lay at the core of the model building phenomenon, was that a model of non-stationary sequence learning would prove sufficient for this purpose. Following this we describe how we were unable to fully capture the range of performance of our participant groups with the original ELPH model, primarily because of a tendency for ELPH to outperform most healthy human subjects regardless of numerical changes to the parameters. We next present results with a variation of ELPH that replaces its preoccupation with predicting future *observations* to instead focus on predicting *choice rewards*; this was done by using a simple reinforcement mechanism. While demonstrably sub-optimal, this combined model better captured the range of performance seen in both patient groups and healthy controls (which were also sub-optimal). We conclude with some conjectures about what forces may have led human cognitive systems to resort to sub-optimal decision making processes.

## Materials and Methods

### 1. RPS Experiment

The data from our previous study [13] form the comparison set for the computational simulations performed here. Three groups of participants (right brain damaged: RBD, left brain damaged: LBD and healthy controls: HC) played 600 trials of RPS against a computer opponent. Participants were not informed that the computer initially followed a strategy of uniform choice for the first 200 trials, followed by a lightly biased choice (200 trials with rock chosen half the time) to finally playing a heavily biased choice (200 trials with paper chosen 80% of the time). Only the last 200 trials (the heavy bias of paper) were used for this study given that no evidence of learning the computer's strategy could be found in any of the groups for the first two conditions (more details are provided in the Text S1; also for full results and procedures see [13]).

### 2. Computational Model

We hypothesized that for our RPS task, humans develop their mental model about the task through generating various hypotheses about what should be played next based on what has been observed so far. Then, the participants constantly update and evaluate these generated hypotheses according to new incoming information. More predictive hypotheses are kept in working memory and are used to predict which action is to be taken next. Less predictive hypotheses are pruned.

Two different mechanisms seemed the most plausible approaches to be taken by our participants in their efforts to beat the computer opponent: (1) they could utilize a statistical learning approach and focus on learning what choice the computer would play next- they would then use this prediction to select their choice-; (2) they could bypass learning to predict their opponent and employ a reinforcement learning approach to learn directly which choice they should make given past choices.

To examine whether either of these approaches were plausibly used by our human participants, we developed two computational models, one based on each approach and compared them for their ability to reproduce the pattern of plays and wins for each participant individually and for the patient group (RBD, LBD, or Control) to which they belonged. The main ideas of hypothesis generation, hypothesis updating, hypothesis evaluation and hypothesis pruning were common to both models; the principal difference between them was whether the hypotheses were about what the opponent would play, or whether the hypothesis was about whether their own choice was likely to win.

#### 1.1. Statistical Learning Approach: ELPH

A sequence learning approach called “ELPH” is used to predict the behaviour of the participants by employing a statistical learning approach [17]. ELPH proposes a two-compartment approach to sequence learning. The Short Term Memory (STM) component contains the *n* most recent temporally ordered observations and the Hypothesis Space (HS) contains individual hypotheses. These hypotheses predict what is likely to be observed given what has already been seen. To learn the best hypothesis, ELPH has three main functions; hypothesis learning, prediction and pruning. Each function is described separately bellow.

#### Hypothesis Learning Function

The ELPH hypothesis generation function consists of two sub-functions; Hypothesis Generation and Hypothesis Updating.

#### Hypothesis Generation

Individual hypotheses in ELPH are ordered subsets of prior observations taken from STM. As an example, imagine that ELPH is exposed to the pattern below:

rock, rock, paper, rock, rock, paper, rock, rock, paper,…

Let's say the length of STM (*n*) is equal to 2, which means at the end of each trial the last two observations are saved in STM. Imagine also that the last two observations (at time t-2 and t-1 respectively) were ‘paper’ and ‘rock’. Then the current content of STM would be the tuple (‘paper’, ‘rock’). The goal is to learn to predict the next observation based on the current content of STM. To do so, ELPH generates hypotheses about the possible relationships between the computer's last plays (which have been saved in STM) and its next play.

The logic behind the hypothesis generation function is that every subset of STM might be a predictor of the next observation. In our example, the strategy might be that playing rock at each trial (t-1) is always followed by playing another rock at the next trial (t) (Hyp_1_). It is also possible that rock is played (at time t) only after playing a paper and a rock respectively (Hyp_2_). The last possible option is that playing paper at t-2 is an indicator of playing rock at t regardless of whatever item appeared at t-1 (Hyp_3_).

#### Hypothesis Updating

The learning process is to learn over time which of these generated hypotheses predicts the next observation best and determine the most likely prediction. To learn the predictive hypotheses, a “prediction-set” is associated with each hypothesis consisting of all the events that have immediately followed that hypothesis and a count of the number of times each event has happened. For a particular hypothesis, Hyp, a prediction-set is as follows:
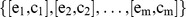
here e_1_, e_2_ … e_m_ is the list of possible outcomes (rock, paper, or scissors in the present case) and c_1_, c_2_… c_m_ represents the counts of how many times each has been observed. To update a prediction-set, after observing new data, the corresponding count (c_i_) of that event of the matching hypothesis (e_i_) is incremented by one. If this prediction has not been previously observed, this event is added to the prediction-set (c = 1). In our example, after 21 trials, the three generated hypotheses will be updated to:
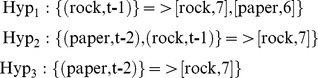



This means the sequence of paper and rock has been observed 7 times so far. While Hyp_2_ and Hyp_3_ always leads to rock, Hyp_1_ has been followed by rock 7 times and by paper 6 times. The idea behind the learning process in this method is that if a subset of observations has been followed consistently by an event, it is most likely to be followed by the same event in the future. Thus, the learning process is simply an updating of these prediction-sets based on incoming data.

Prediction Function: To make a prediction from these hypotheses and their sets of observations, we need to select the best hypotheses whose predictions are most consistent and reliable or, more accurately, less random. In our example, in contrast with Hyp_2_ and Hyp_3_, Hyp_1_ has less predictive value; because it is followed half of the time by paper and half of the time by rock. To mathematically evaluate how trustworthy the predictions of a hypothesis are, a modification of the entropy of each hypothesis is calculated at each trial [18]. For a particular hypothesis, Hyp, with a prediction-set of {[e_1_, c_1_], [e_2_, c_2_]… [e_m_, c_m_]}, the modified entropy is calculated below:
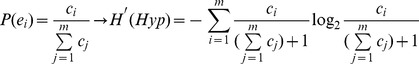
(1)


Among all related hypotheses, the most predictive one is the one with the lowest amount of entropy. The probability of selecting a hypothesis to predict the next observation is proportional to its entropy. The lower the entropy of a hypothesis, the more probable for that hypothesis to be selected. After selecting the proper hypothesis, each event in the prediction-set of this hypothesis (e_k_, k = 1,…, m) has a probability proportionate with the count of that event (denoted by c_j_ for each e_j_) to be chosen as the prediction of the computer's next play (mathematical formulation is provided in Text S2).

Pruning Function: Prediction in ELPH requires recording all the items that have been observed so far. There are two principal problems with simply accumulating all past observations. First, the number of hypotheses grows too large, too quickly, as STM capacity increases. If *n* equalled 5, there would be 2^5^-1 related hypotheses stored in HS. The second problem is that the history of observations lengthens. To compensate for this growth in the hypothesis space, the inconsistent hypotheses with conflicting predictions are removed from HS. The entropy measure is used again to calculate the amount of randomness associated with individual hypotheses. Hypotheses with an entropy value more than a given threshold (denoted as H_thr_) are deleted at the end of each trial.

In summary, at the beginning of each trial, the hypothesis learning function creates all the possible subsets of the current content of STM as potentially new hypotheses. Generated hypotheses may either exist in HS or may be novel. To make a prediction about the next observation, the prediction function examines HS to search which hypothesis already exist in HS. Those that already are in HS are used to make a prediction. After the prediction, new data is observed and the hypothesis learning function updates all the related hypotheses according to this new data. The generated hypotheses which were not in the HS before are then augmented. The prediction-set for those hypotheses would consist of this new event (observation) followed by the event count set to 1. At the end, the pruning function calculates the entropy of all the existing hypotheses in HS and deletes those with entropy value exceeding a given threshold (for more information see Text S2).

#### 1.1. Reinforcement learning Approach: RELPH

While ELPH predicts the computer's next play, RELPH is a reinforcement learning based approach which learns the most rewarding item. The main difference between ELPH and RELPH lies in the prediction function. Just as is the case for ELPH, RELPH consists of three main functions: hypothesis learning, prediction and pruning. Where ELPH employs the most predictive hypotheses to predict the next most likely *observation*, RELPH uses *the most rewarding hypotheses* to select the best item to be played in subsequent trial. The pruning function, however, stays the same; non-predictive hypotheses are deleted from HS to facilitate adaptation. These three functions of RELPH are described below.

#### Hypothesis Learning Function

As in ELPH, the RELPH hypothesis generation function has two main sub-functions: Hypothesis Generation and Hypothesis Updating.

#### Hypothesis Generation

The main difference between the hypothesis generation function in RELPH and ELPH is that instead of opponent's observations being stored, it is RELPH's choices that are stored in the prediction-set. Indeed in ELPH, hypotheses are generated to answer the question of which *item* is most likely to be observed next. In RELPH, the question is different. Hypotheses represent which choice is most likely to *lead to winning* the next trial. Thus in RELPH a prediction-set is defined as all the items that the RELPH player has tried followed by the total amount of reward RELPH received for playing each of those items. Each win was set to a reward value of +1 for a win, a tie to 0 and a loss to −1. As a result, for a particular hypothesis, Hyp, a prediction-set is defined as follows:
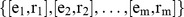
in which e_1_, e_2_ … e_m_ is the list of plays (rock, paper, or scissors in the present case) and r_1_, r_2_… r_m_ represents the total amount of reward gained for playing each item. The learning task is to learn the most rewarding hypothesis. To do so, prediction-sets of hypotheses should be updated based on incoming data.

#### Hypothesis Updating

Hypothesis updating includes two steps: prediction-set updating and value updating. Prediction-set updating refers to updating each hypothesis's prediction-set according to the result of the last play. The rule stays the same; if the hypothesis generated based on the current content of STM already exists in HS, its prediction-set will be updated. If not, this hypothesis will be added to the HS. For the existing hypotheses, if the last play already existed in the prediction-set, the corresponding total reward is updated based on the last value of reward. If not, this new play and the associated reward value are added to the prediction-set. In our example, (for *n* = 2 and STM content of the tuple (‘paper’, ‘rock’)), if RELPH played ‘scissors’ at the next trial, it would lose to the computer when the computer plays rock. In this case, the generated hypotheses would be:
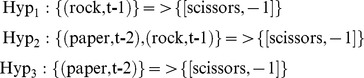
if the next time that the RELPH player observed a sequence of paper and rock, it played paper then the three above hypotheses would update to:
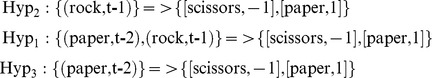
Based on these prediction-sets, it is clear that between scissors and paper, paper is the more rewarding option to play next time.

Value updating refers to updating the value of each hypothesis based on the last reward value. To find the most rewarding hypothesis, a value function is defined over the hypothesis space. The value of each hypothesis is the expected reward that the learner receives by playing that hypothesis To learn the value of each hypothesis, the initial values are set randomly and are updated at the end of each trial after taking an action and according to the delta-learning rule [19]:
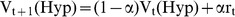
(2)


V in this formula refers to the value of hypothesis Hyp, r_t_ to the instantaneous reward at time t and α refers to the learning rate, the parameter adjusting the weight of previous estimation of the hypothesis value and the instantaneous reward. The closer this parameter is to one, the more significant the role of instantaneous reward is in determining the hypothesis value and vice versa; the smaller the value of α, the greater the role of prior reward.

#### Prediction Function

The most eligible hypothesis for prediction in RELPH is the most rewarding one. Yet to balance exploration and exploitation we used the soft-max decision rule [20], [21], [22], [23] as it has been commonly used for this purpose [24], [25].

#### Pruning Function

The idea behind the pruning function in RELPH is similar to ELPH. Instead of event entropy, outcome entropy is computed (for mathematical formulation of action-selection rule and pruning function see Text S2).

### 2. Parameter Estimation and Model Comparison

Models “played” against the same sequences of rock, paper, and scissors choices as the computer opponent made during our behavioural study [13]. Although only the result for the last 200 trials (where the computer opponent's choices were heavily biased toward paper) is presented in the results part of this paper, it is important to mention that both ELPH and RELPH were exposed to the exact same sequence of play that our participants saw from the very beginning to end (trial 1–600). For each human participant we created a matched ELPH/RELPH model. To find the best-matched version of each model, we needed to find the parameter set that most closely approximated each individual participant. ELPH has two parameters; the length of STM, denoted by *n*, and the entropy threshold for the pruning function, denoted by H_thr_. In addition to these parameters, RELPH has an extra parameter; learning rate (α). Best parameter sets were computed using maximum likelihood (ML) estimation [26], [27].

There are several methods suggested to find the optimal parameters in ML estimations. Due to the peculiar characteristic of this model not having a specific input-output mapping, traditional optimization methods were not applicable. To overcome this challenge, we searched through a lattice of possible values for the parameters that resulted in ELPH playing the sequence of choices that a participant had played. Best parameter sets were those that resulted in ELPH (RELPH) most closely replicating the sequence of choices made by a particular participant.

To evaluate model performance we calculated win rates. To assess differences and changes between the performance of each model and our participants, we employed repeated measures analyses with the within subjects factors of time (trials divided into twenty blocks of 10) and a between subjects factor of player (Human vs. Computer model). These analyses were done separately for ELPH and RELPH. In addition, to compare the fitness of our models, we calculate both Bayes factor [28] and Akaike's information criteria (AIC; [29]).

## Results

### 1. Healthy Controls and ELPH

**Figure 1:**
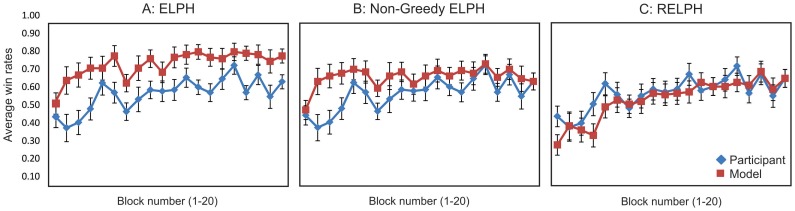
The average win rate of HCs versus ELPH and RELPH. Each plot shows the average win rate over the last 200 trials in the RPS experiment of Danckert et al. [13] for HCs versus (A) ELPH, (B) non-greedy ELPH and (C) RELPH. The blue line represents the average win rate of HCs. The red line shows the average win rate for the (A) ELPH, (B) non-greedy ELPH and (C) RELPH. Error bars represent the standard error of the mean.

The repeated measures analysis demonstrated a significant main effect for time (F (19, 418) = 3.36, p<.001, η^2^ = .13), with a marginally significant difference between players (F (1, 22) = 3.86, p = .062, η^2^ = .15) for ELPH compared to HCs. Although there was no difference between players for the very first 10 trials, ELPH significantly outperforms HCs between trials 11–40. From trial 41 onward there was no significant difference between HCs and ELPH (all p's>.05; Figure 1.B). This means that while ELPH matches HCs for win rate at the end of the block, it reaches this performance sooner (i.e. it learns faster than our participants).

### 2. Healthy Controls and RELPH

The same analysis for RELPH and HC only showed a significant main effect for time (F (19,418) = 6.92, p<.001, η^2^ = .24). Both RELPH and human players increased their win rates over the 200 trials with no significant difference between them (F (1, 22) = .50, p>.45, η^2^ = .02), and no difference in the rate of learning (no significant interaction between time x player (F (19, 418) = .78, p>.70, η^2^ = .03). Even within the first 20 trials there was no difference between the performance of RELPH and HCs (all p's>.05) (Figure 1.C)

Comparing the result of RELPH and ELPH suggests that RELPH is more successful at capturing the behaviour of the healthy participants. To statistically compare the fitness of these two models, AIC is calculated for each model (see Table 1). The result shows that RELPH describes the data better than ELPH. To demonstrate how significant this different is Bayes factor is computed (see Table 1). Clearly RELPH better replicated the result of HCs compared to ELPH, suggesting that the type of learning is the critical factor to model human performance in the RPS task. Whereas ELPH learns the probability of observing each option and selects the most probable option, RELPH learns the association between each option and the estimated amount of reward for playing that option; consequently RELPH plays the most rewarding action.

**Table 1:** AIC value and Bayes factor computed for each model (ELPH and RELPH) per each group separately.

	AIC(ELPH)	AIC(RELPH)	2ln(k)
HCs	314.428	177.185	35.862>10
RBDs	391.204	285.287	159.318>10
LBDs	326.959	188.297	232.203>10

Bayes factor is calculated as 2ln(k) in which 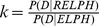. D in this formula is the observed data which in our case is the participants' sequence of plays.

### 3. LBDs and RELPH

**Figure 2:**
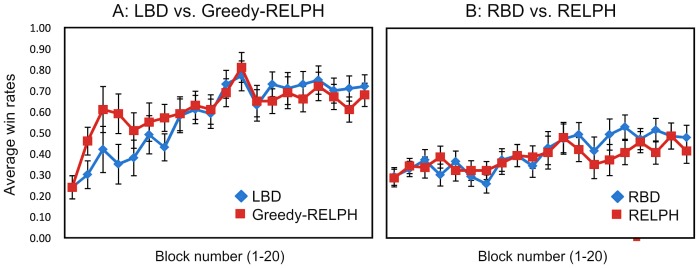
The average win rate for patient groups versus RELPH. Each plot represents the average win rate for (A) greedy-RELPH and (B) RELPH, (red lines) against the average win rate of (A) LBD, (B) RBD patients (blue lines) over the last 200 trials in the RPS experiment of Danckert et al. [13]. Error bars represent the standard error of the mean.

Inspection of Figure 2.A demonstrates that LBD participants not only solved the RPS task as well as HC, in many cases they exceeded the performance of HCs as they played long runs of the best choice (data not shown here; see Figure 5 in [13]). This strategy is one of probability maximization. This observation suggests that the best way to get RELPH to approximate LBD performance is to “turn-off” the soft-max choice rule. Indeed this ‘greedy’ version of RELPH, with all other parameters unchanged (compared to HCs) was able to replicate the result of LBD patients. LBD patients and the greedy-RELPH algorithm increased their win rates over the course of the 200 trials (F (19, 342) = 9.05, p<.001, η^2^ = .33) with no significant difference between RELPH and LBDs player (F (1, 18) = .35, p>.55, η^2^ = .02) or learning rate (no interaction between time x player: F (19, 342) = 1.07, p>.35, η^2^ = .06). There was no significant difference in the proportion of win rates between LBD and greedy-RELPH at any time point (all p's>.05) (Figure 2.A).

To make sure the difference between LBDs and HCs are only due to the soft-max compartment, not the type of learning, the AIC value and Bayes factor for both ELPH and RELPH are outlined in Table 1. Bayes factor is greater than 10 representing the fact that the difference between these two models is highly significant [28].

### 4. RBDs and RELPH

RELPH was also successful at replicating the result of RBDs. RBDs and RELPH both significantly increased win rates over time (F (19, 494) = 3.87, p<.001, η^2^ = .13) with no significant main effect for player (F (1, 26) = .21, p>.60, η^2^ = .01) or significant interaction between player x time (F (19, 494) = .76, p>.75, η^2^ = .03). At no point over the 20 intervals of the last 200 trials was there a significant difference between win rates of RBD patients and RELPH (Figure 2.B). The AIC value for adjusted ELPH and RELPH as well as Bayes factor are presented at Table 1. RELPH is again significantly better at capturing the performance of RBDs comparing to ELPH.

Clearly, the HC and RBD groups performed differently with the latter failing to fully exploit the biased play of the computer opponent. Nevertheless, RELPH was able to replicate the performance of both groups. To better understand RELPH's ability to model both HC and RBD participants, we looked at the parameter values that resulted from the matching procedure employed (Table 2 and Table 3).

**Table 2:** The mean (and standard deviation) of each parameter for RBDs vs. HCs.

	HC	RBD	T	p-value
H_thr_	2.28 (0.82)	1.72 (0.89)	1.67	0.11
α	0.27 (0.22)	0.29 (0.23)	−0.28	>.75

**Table 3:** Frequency of *n* (length of STM) for HCs and RBDs.

*n*	HC	RBD
1	2	3
2	7	8
3	3	3

The average of the best-matched parameters for each group is displayed in Table 2. Although neither H_thr_ nor α revealed a significant difference between groups, RBD patients tended to have a lower H_thr_ compared to HCs. We did not find any difference in optimal value of *n* (length of STM) used by HCs and RBD patients (Likelihood ratio = .114, df = 2, p>.90) (Table 3).

Although group performance of the RBD patients in [13] was poor, there were exceptions (see Figures 4 and 5 in [13]). Therefore, we compared the RELPH parameters fit to RBD patients who did manage to exploit the strong bias to some degree (defined as above chance performance on the last 100 trials; n = 6) to the majority of RBD patients that did not (n = 8). RELPH fits for RBD patients who played above chance compared with those who did not tended to have a higher H_thr_ (Mean = 2.20, SD = .38 vs. Mean H_thr_ = 1.35, SD = 1.01; t(12) = 1.01, p = .057) and a lower α (Mean = .12, SD = .14 vs. Mean α = .42, SD = .20; t(12) = 3.09, p<.01). No significant difference was found for *n* values.

## Discussion

Our interest in computational models derives from our interest in brain damaged patients. We recently advanced the idea that impairments in building mental models and updating these models may provide an explanation for the heterogeneous impairments that follow RBD [30], [16]. To support these claims we examined the performance of both brain damaged patients and controls playing RPS against a computer that could employ a variety of strategies and switched among them [31], [13].

One intuitive idea for how it is that humans learn to master sequential tasks is that they learn to predict what will happen next in a sequence. The final goal for humans in a sequential decision making task is to learn what action is optimal to take at each step. To learn those actions they learn to make a prediction about what will happen next, based on the history of observations and then according to this prediction the best action is taken. In other words, humans treat those tasks as statistical learning tasks in which they are supposed to detect the regularities that exist in the sequence and use those data to predict the next observation (for reviews on statistical learning see [32], [33]). This characterization of the process gives priority to predicting observations. This emphasis seems sensible because one optimal way to figure out what action must be taken next is, of course, to figure out what will happen next. In addition, the statistical learning impairment reported in this patient population is another reason to believe that this type of learning might be involved in the sequential decision making impairment seen in brain damaged patients [34]. These reasons encouraged us to use a sequence learning method, the ELPH model [17], for our computational studies.

What the computational modelling study revealed though, was that learning to predict what will happen next did not provide a good match to what our human participants were doing, whether or not they had brain damage. Based on our results, there is a main problem with statistical learning; ELPH learns faster than humans for all three groups of participants. In all cases the ELPH model, even when hobbled by extra memory constraints and a choice selection rule (which were not part of the original model's specifications; [17]), outperformed all our participant groups by learning to exploit the 80% bias more quickly than human learners. Performance parity only occurred when we changed the ELPH model to associate choices with reward, and to focus on learning how rewarding particular hypotheses were.

In the RELPH model instead of learning the regularities to predict the next observation and then deciding on the action, the best action was learned directly based on the previous experience the model had for taking that action. Based on this model, the best action to take now is the action that was most rewarding when the model faced the same situation in the past. This hybrid version, which we call RELPH, captured the performance of both HCs and RBDs patients. It was also capable of capturing the performance of LBD participants when we introduce a coexistent ‘lesion’ of the soft-max choice component. The interim conclusion is that it is more likely that learning to associate actions with rewards in a context specific fashion mediates performance more strongly than simply learning to guess the next token in a sequence, despite that fact that it seems intuitively better to do so (and despite the superior performance of the ELPH model version).

Why is it the case that we would use this sub-optimal strategy? It may reflect the general complexity of the task. In real life situations, to solve a learning problem it is often necessary to solve several sub-problems in parallel. One of the most computationally demanding of the sub-problems is learning how to generate good hypotheses. To make good hypotheses, all the features of the environment that could impact our decisions must be known, identified, and tracked. In most real-world scenarios this relevant feature-space is large, unknown, and commonly computationally intractable [35]. This source of complexity however is totally ignored in the ELPH model since the model is “told” what the relevant features are, and is built to attend to them. Given the inherent limitations of human information processing, employing sub-optimal but tractable solutions may be rational [2]. As an example of a sub-optimal solution, humans may employ experience-based approaches that focus on what they did and what resulted. This is consistent with decades of research in behaviorist psychology [19] and more modern work applying reinforcement learning algorithms to human performance [24],[36].

These results also have implications for our understanding of the basis for differing performances across patient groups. The results of LBD patients and HCs indicate that they both learn a strategy that results in win rates better than chance. While HCs typically show a behaviour known as probability matching (i.e., they play the optimal option in proportion with its probability of being selected; for a review see [37]), the majority of LBD patients played as maximizers (i.e., choosing the best option almost all the time; see Figure 5 in [13]; for a discussion on possible reasons for choosing a maximizing strategy in LBD patients see page 2756 in [13]). Our computational results suggests that the reason behind this discrepancy is due to different exploitation strategies. In order to learn the best option in experienced-based approaches such as RELPH, all the options must be explored. In our model, exploration is required to make sure that the real value of each hypothesis is learned. Otherwise, we might stick with a poorly rewarded hypothesis simply because we did not explore other options. Of course, trying hypotheses other than the currently “best” one runs the risk of obtaining a lower reward rate. In the strategy used by the computer opponent in the RPS game there was no particular benefit to exploration, only costs. In this narrow realm, always exploiting leads to better performance for the “greedy” versions of the computational models, but this benefit would not be true in general.

Parameter fits for the patient groups also suggested another basis for group differences. Comparing RELPH results for those RBD patients who failed to exploit an opponent's bias with those who showed some exploitation, indicated lower H_thr_ values and higher α values for the participants with poorer performance. α is the parameter which tunes the relative importance of instantaneous reward. The lower the parameter, the less emphasis that is placed on immediate reward. H_thr_ on the other hand, represents the level of noise or randomness that is expected. The higher the H_thr_ the more tolerance to error in the hypotheses prediction. In combination, this suggests that RBD patients explored less efficiently; compared to HCs, they were looking for more certainty in their hypotheses. Meanwhile they placed greater weight on immediate rewards and as such, losing could affect their beliefs about the value of hypotheses more readily. Putting these together, our model suggests that RBD patients would give up on a given hypothesis sooner than what they should. Since they want more predictive hypotheses, and they are highly sensitive to losses, they would switch more often from one hypothesis to another without fully exploiting the available information. This poor exploration strategy led to impaired learning of the computer's strategy.

In summary, we compared two versions of a computational model: one based on statistical learning (ELPH) and another based on reinforcement learning (RELPH). Our result suggests that regardless of the presence of brain-damage, participants learn the action-reward association rather than learning the regularities in the sequence, even though this would have led to better performance. Our model also suggests that what distinguishes LBDs, RBDs and HCs from each other is not different types of learning, but different exploration-exploitation policies. LBDs don't explore as much as HCs, and RBDs explore less efficiently or even too much.

## Supporting Information

Text S1 **The Experiment Procedure.**
(DOCX)

Text S2 **Mathematical Formulas.**
(DOCX)
