# Supplementary material for: Sequential Decisions: A Computational Comparison of Observational and Reinforcement Accounts
Source: PLoS One. 2014 Apr 18;9(4):e94308. doi: 10.1371/journal.pone.0094308 (PMC3991603; doi:10.1371/journal.pone.0094308)
Supplement: Text S1 — The Experiment Procedure. (DOCX) [file pone.0094308.s001.docx]

S_1_. The Experiment Procedure:

In our RPS study each trial began with 2 red squares, vertically aligned in the center of the screen; one representing the computer’s choice and another representing the participant’s choice. Participants were told that once the computer made its choice on a given trial, the square representing the computer would change from red to green and then stay fixed until the end of the trial. Participants were then free to make their own choices and change their choices until they reported that their selection was finalized. After that, the computer’s choice would be revealed. When the participant was ready, another button press began a new trial. No explicit feedback was given regarding the outcome of a given trial.

In this experiment, participants played 600 rounds of RPS against a computer starting with a “random condition” in which each item (i.e. ‘rock’, ‘paper’ or ‘scissors’) had the same probability of being chosen by the computer. After 200 trials, the computer strategy switched to a light bias of ‘rock’ for another 200 trial in which rock was chosen half of the time. Each of the two other options had 25% chance of being picked. Finally, for the last 200 trials the computer strategy switched to the ‘strong’ condition, in which paper was played 80% of the time and the two other options had the same probability of 10% to be chosen.
