# Supplementary material for: Sequential Decisions: A Computational Comparison of Observational and Reinforcement Accounts
Source: PLoS One. 2014 Apr 18;9(4):e94308. doi: 10.1371/journal.pone.0094308 (PMC3991603; doi:10.1371/journal.pone.0094308)
Supplement: Text S2 — Mathematical Formulas. (DOCX) [file pone.0094308.s002.docx]

S_2_. Mathematical Formulas:

- Hypothesis-selection rule in ELPH: probability of selecting Hyp_i_ is proportional to its entropy:

$$\text{P(Hyp}_{\text{i}}\text{)=}\frac{\text{exp(-H(Hyp}_{\text{i}}))}{\sum_{\text{j}} \text{exp(-H(Hyp}_{\text{j}}\text{))}}$$

- Choice-selection rule in ELPH: probability of selecting each e_i_ in the prediction-set of Hyp is proportional to its count (c_i_):

$$\text{P(e}_{\text{i}}\text{)=}\frac{\text{c}_{\text{i}}}{\sum_{\text{j}=1}^{3} \text{c}_{\text{j}}}$$

- Hypothesis-selection rule in RELPH: probability of selecting Hyp_i_ is proportional to its value (known as soft-max formula):

$$\text{P(Hyp}_{\text{i}}\text{)=}\frac{\text{exp(V(Hyp}_{\text{i}}\text{))}}{\sum_{\text{j}} \text{exp(V(Hyp}_{\text{j}}\text{))}}$$

- Choice-selection rule in RELPH: probability of selecting each e_i_ in the prediction-set of Hyp is proportional to its accumulated reward (known as soft-max formula):

$$\text{P(e}_{\text{i}}\text{)=}\frac{\text{exp(r}_{\text{i}}\text{)}}{\sum_{\text{j}=1}^{3} \text{exp(r}_{\text{j}}\text{)}}$$

- Entropy formula in ELPH: the event entropy value for hypothesis, Hyp, is calculated bellow:

$$\text{H(Hyp)= -}\sum_{\text{i}=1}^{3} \frac{\text{c}_{i}}{\sum_{\text{j}=1}^{3} \text{c}_{\text{j}}}\log_{2}\frac{c_{i}}{\sum_{\text{j}=1}^{3} \text{c}_{\text{j}}}$$

- Entropy formula in RELPH: the outcome entropy value for hypothesis, Hyp, is calculated bellow:

$$\text{P(o}_{\text{i}}\text{ )=}\frac{\text{o}_{\text{i}}}{\sum_{\text{j}=1}^{3} o_{j}}\to\text{H(Hyp)=-}\sum_{\text{i}=1}^{3} \frac{\text{o}_{\text{i}}}{\sum_{\text{j}=1}^{3} o_{j}}\log_{2}\frac{\text{o}_{\text{i}}}{\sum_{\text{j}=1}^{3} o_{j}}$$

in which o_i_ is the count of numbers that win, tie and lose have happened.
